# Large-Scale Predictions of Compound Potency with Original and Modified Activity Classes Reveal General Prediction Characteristics and Intrinsic Limitations of Conventional Benchmarking Calculations

**DOI:** 10.3390/ph16040530

**Published:** 2023-04-02

**Authors:** Tiago Janela, Jürgen Bajorath

**Affiliations:** Department of Life Science Informatics and Data Science, B-IT, LIMES Program Unit Chemical Biology and Medicinal Chemistry, Rheinische Friedrich-Wilhelms-Universität, Friedrich-Hirzebruch-Allee 6, D-53115 Bonn, Germany; janela@bit.uni-bonn.de

**Keywords:** compound potency predictions, activity classes, machine learning, nearest neighbor controls, benchmark calculations

## Abstract

Predicting compound potency is a major task in computational medicinal chemistry, for which machine learning is often applied. This study systematically predicted compound potency values for 367 target-based compound activity classes from medicinal chemistry using a preferred machine learning approach and simple control methods. The predictions produced unexpectedly similar results for different classes and comparably high accuracy for machine learning and simple control models. Based on these findings, the influence of different data set modifications on relative prediction accuracies was explored, including potency range balancing, removal of nearest neighbors, and analog series-based compound partitioning. The predictions were surprisingly resistant to these modifications, leading to only small error margin increases. These findings also show that conventional benchmark settings are unsuitable for directly comparing potency prediction methods.

## 1. Introduction

Compound potency prediction is of major interest in medicinal chemistry and drug design. Many different computational methods have been introduced for potency predictions based on structures of ligand-target complexes or small molecules [1,2,3,4,5,6,7,8,9,10,11]. These approaches have different computational complexity and sophistication. Traditionally, quantitative structure–activity relationship (QSAR) methods have played a major role in medicinal chemistry [1]. Classical QSAR models are based on two-dimensional representations of small molecules, typically employ numerical descriptors of molecular structure and chemical properties, and represent linear regression models to predict the potency of newly designed compounds to extend analog series. Thus, QSAR only applies to congeneric compounds if linear structure–activity relationships (SARs) exist [1]. In addition, for the estimation of interaction energies from experimental or modeled protein–ligand complexes, a variety of scoring functions were developed that are, for the most part, based on force fields from molecular mechanics [2]. Estimating interaction energies using scoring functions of different designs and complexity is used as a rough approximation of binding (free) energies and relative potencies of other ligands (without calculating exact potency values). Scoring functions apply to diverse compounds and are critically important to prioritize putative ligands from structure-based virtual screening, despite their approximate nature [2]. At a higher level of sophistication, free energy methods attempt to calculate exact binding free energy values from protein–ligand complexes based on thermodynamic principles [3]. Particularly popular in medicinal chemistry and drug design are free energy perturbation methods to calculate relative binding free energies of congeneric compounds based on molecular dynamics simulations by “alchemically” transforming one analog into another. Compared to force field calculations, relative free energy calculations are computationally very expensive. Although free energy methods have been available for three or four decades, they have been increasingly applied in recent years in drug discovery due to advances in computational power and conformational sampling procedures [3]. 

Furthermore, in structure-based design, binding energy, and compound potency values can also be calculated using methods that combine molecular mechanics (MM) treatment of protein–ligand complexes with quantum mechanical (QM) representations of narrowly defined ligand binding sites (termed QM/MM approaches) [4]. The underlying idea is achieving accurate energy calculations in binding sites through quantum mechanics while reducing computational costs for the remainder of complexes to render the calculations feasible. For ligand-based potency prediction, machine learning (ML) methods play a major role [5,6,7]. Therefore, suitable ML methods must be applicable for regression. Compared to QSAR, the major attraction of computationally more complex ML approaches is their ability to account for non-linear SARs and predict potency values of structurally diverse compounds. Non-linear SARs are typically observed in medicinal chemistry when optimizing compound series, which intrinsically limits the applicability domain of classical QSAR. Accordingly, ML regression models have become very popular for compound potency prediction. The majority of approaches include ML mainstay methods such as random forest regression [6] or support vector regression (SVR) [7,8]. Over the years, SVR has become the probably most frequently used ML approach for numerical potency prediction and a standard in the field. Recently, deep neural networks (DNNs) have also been increasingly applied for this task [9,10,11]. Many different DNN architectures can be adapted for numerical property predictions, including compound potency. This methodological versatility is a major attraction of DNNs [9,10,11]. Moreover, DNNs enable the evaluation of new concepts for potency prediction. For example, convolutional neural networks can predict numerical properties from voxel representations of ligand binding sites. For chemical applications, graph neural networks have become increasingly popular, and they have also been adapted for ligand affinity predictions. Therefore, graph representations of molecular interactions are extracted from structures of protein–ligand complexes and used as input for deep graph neural networks to predict the affinity of small molecular ligands. Exploring novel concepts for potency predictions is still in its early stages (and some findings are controversial). Hence, it will take time until these approaches mature. While DNN calculations are computationally much more expensive compared to other ML approaches, they are not necessarily superior for potency prediction [12], as further discussed below.

The prediction of compound potency (and other biological or physico-chemical molecular properties) is carried out to benchmark or calibrate computational approaches and, in addition, prospectively predict novel active compounds. While prospective applications are naturally most interesting in medicinal chemistry and drug discovery, benchmarking is essential for the initial evaluation of predictive models but insufficient to ensure successful applications. Typical benchmark conditions for numerical potency prediction involve using sets of specific active compounds (often termed activity classes) with varying potency divided into training sets for model derivation and test sets for evaluation, usually with cross-validation on the basis of multiple independent prediction trials. Analogous benchmark settings are applied to assess compound classification models (derived, for example, to distinguish between active and inactive compounds).

Recently, we have shown that potency prediction methods of varying computational complexity display similar predictive performance [12]. Specifically, k-nearest neighbor (kNN) analysis was found to reproduce experimental potency values of test compounds within an order of magnitude comparable to increasingly complex ML methods, including DNNs. In 1-NN analysis, test compounds are compared to training set compounds via similarity calculations, and the potency value of the most similar training compound is assigned to a given test compound. For 10 different activity classes, there was no advantage of DNNs over SVR and kNN calculations, with SVR achieving the overall best performance, albeit by only small margins [12]. Hence, simple predictions were often as accurate as increasingly complex ML methods. Furthermore, assigning the median potency value of a training set to any test compound, corresponding to median regression (MR), often approached the accuracy of ML models. Moreover, randomized prediction models often reproduced experimental potency values within an order of magnitude, and there was only a confined prediction error interval into which random and ML predictions fell.

Questions raised by these observations included whether these findings might generalize across large numbers of activity classes and whether their composition and/or potency ranges might limit benchmarking evaluations. Therefore, in this study, we have systematically investigated compound potency predictions on an unprecedentedly large scale and designed specific data set modifications to investigate their influence on the prediction accuracy of different reference methods. Potency predictions were surprisingly stable across hundreds of compound classes, and relative method performance was largely resistant to specific data set modifications. Furthermore, predictions using ML and simple control models were only distinguished by small error margins, revealing intrinsic limitations of conventional benchmark calculations.

## 2. Results

### 2.1. Study Concept

First, we aimed to obtain a global view of potency prediction characteristics and relative accuracies of selected methods. Therefore, we carried out systematic potency value predictions on 376 qualifying activity classes from medicinal chemistry sources [13] using SVR and controls, including 1-NN, additional kNN, and MR calculations (see Methods). The activity classes were curated, ensuring high-confidence potency data were available for all compounds. SVR was selected as the overall preferred ML approach in our previous comparison [12]. Second, based on the obtained results, we then investigated the influence of specific data set modifications on relative prediction accuracies.

### 2.2. Large-Scale Predictions

Predictions were assessed by calculating the mean absolute error (MAE) for predicted and experimental logarithmic potency values (see Section 4). Given the very large number of activity classes and calculations, all results are made available in a data deposition via the following link: https://uni-bonn.sciebo.de/s/vU5vnG5wjQPTpd1 (accessed on 28 March 2023)). In addition, for representative subsets of activity classes, results are reported in the following and as Supplementary Materials.

For the 376 activity classes, the results of the predictions were surprisingly similar. While MAE values varied moderately across different classes, it was generally observed that 1-NN/kNN predictions approached or met SVR performance, consistent with our earlier observations for 10 activity classes [12]. In addition, most predictions produced meaningful results, with median MAE values over multiple independent trials falling within one order of magnitude, corresponding to less than 10-fold prediction error. Notably, for best predictions, MAE values of 1 or larger were not observed for any activity class. Supplementary Figure S1 shows the results for the 45 largest activity classes that were representative of all 376 activity classes. Hence, only limited class-dependent differences were detected.

Supplementary Figure S1a,b compare predictions for the 45 activity classes based upon 80/20% and 50/50% training/test compound splits, respectively. Again, these predictions yielded very similar results. Hence, different training set sizes had little influence on the predictions. Thus, the predictions were stable, as indicated by narrow MAE value distributions across different trials.

**Figure 1:**
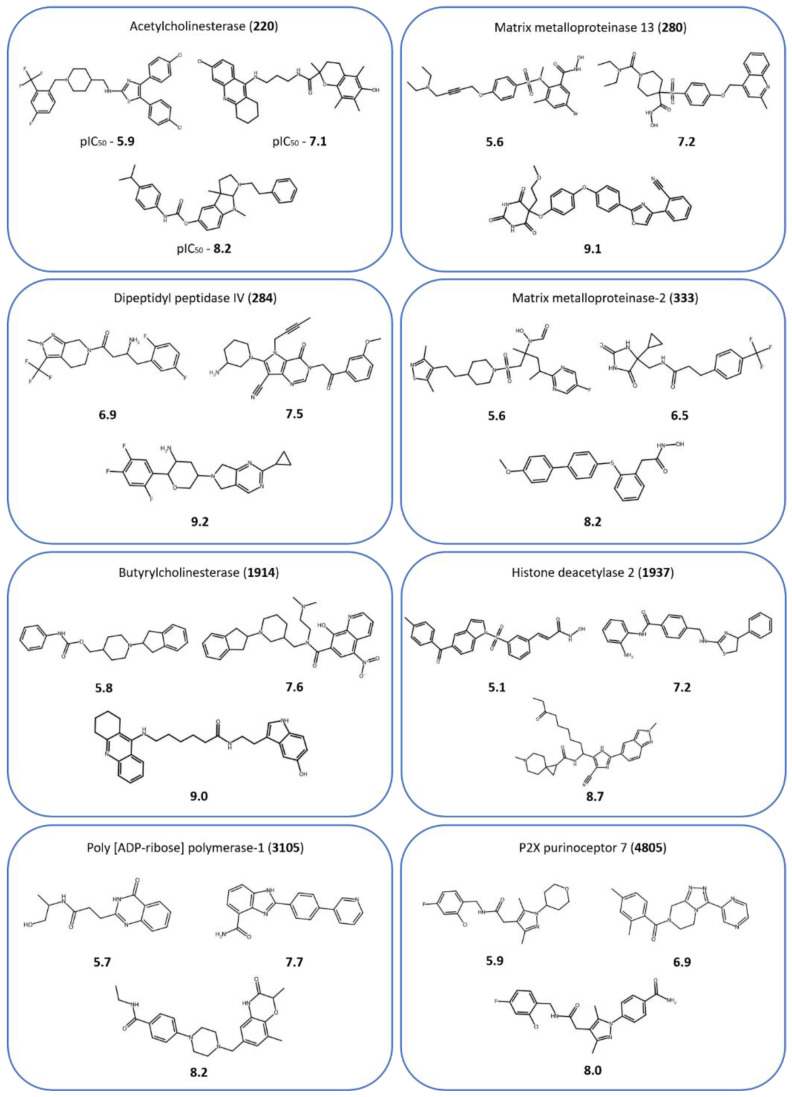
Compounds from selected activity classes. For eight activity classes, exemplary compounds are shown with their logarithmic potency values (pIC_50_). For each class, the target name and ChEMBL ID (in parentheses) are provided.

**Figure 2:**
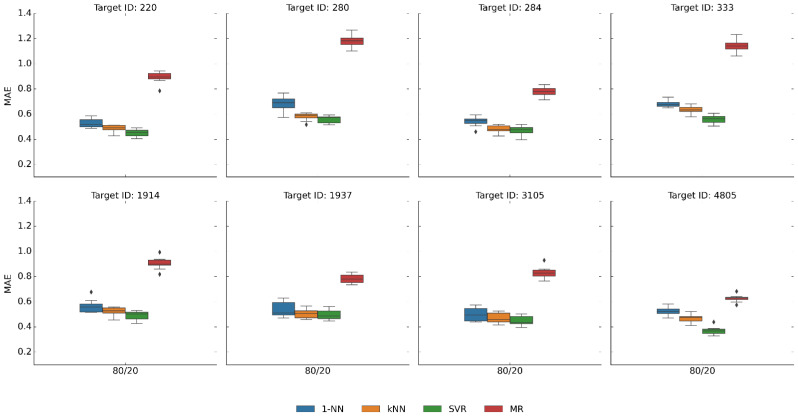
Prediction accuracy. Boxplots report the distribution of MAE values for potency predictions over 10 independent trials on the eight activity classes in Figure 1 using 1NN, kNN, SVR, and MR models (applying a training/test set compound split of 80:20%). In boxplots, the upper and lower whiskers indicate maximum and minimum values, the boundaries of the box represent the upper and lower quartiles, values classified as statistical outliers are shown as diamonds, and the median value is indicated by a horizontal line.

Figure 1 shows exemplary compounds from eight of the 45 activity classes (and reports their targets) used in the following to illustrate results obtained for the 45 largest classes. In addition, Figure 2 shows the results of the original predictions for the eight activity classes and 80/20% splits, illustrating trends commonly observed for all classes. Although SVR mostly achieved the highest accuracy (lowest MAE values), followed by kNN/1-NN, the differences between median values were typically only very small, ~0.1 MAE or even less. Statistically significant differences were only observed for about half of the classes (Wilcoxon test, *p*-value < 0.005; see Section 4). Even the simplistic MR prediction, assigning the constant median potency value of the training set to all test compounds, typically yielded prediction accuracies close to 1.0 MAE. Thus, these findings revealed that (i) even simple control predictions generally produced fairly accurate results and that (ii) there was no sufficient separation between SVR and kNN or MR controls to enable a realistic assessment of ML potency prediction methods. Across as many as 376 different activity classes, essentially no cases were detected where prediction accuracy was low and simple controls failed compared to SVR.

These findings raised the question of whether the activity classes could be modified in specific ways to increase the prediction accuracy separation of SVR and the kNN controls and hence obtain an improved basis for methodological comparisons. These modifications altered the original composition of activity classes by design, thus producing model data sets. The predictions were then repeated on the resulting variants of the 45 largest activity classes. The following shows representative results for the subset of eight activity classes.

### 2.3. Potency Range Balancing

**Figure 3:**
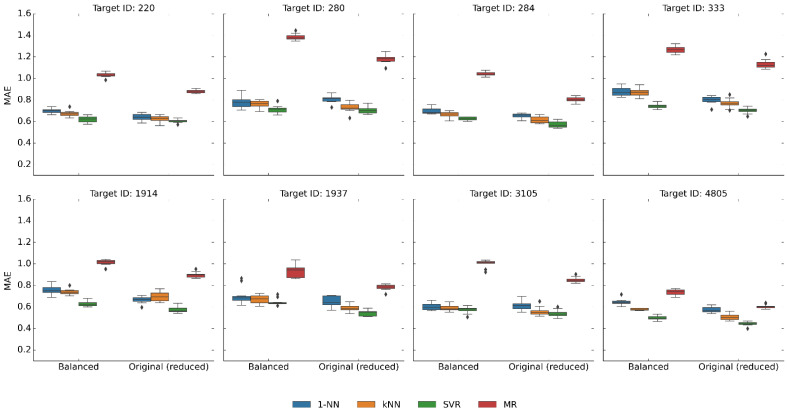
Prediction accuracy for activity classes with balanced potency value distributions. Boxplots report the distribution of MAE values over 10 independent trials for the eight activity classes after balancing their potency value distributions. As a control, results are reported for the original data sets that were reduced by random compound removal to the same size as the balanced sets. In boxplots, the upper and lower whiskers indicate maximum and minimum values, the boundaries of the box represent the upper and lower quartiles, values classified as statistical outliers are shown as diamonds, and the median value is indicated by a horizontal line.

We first determined the potency value distributions across the largest activity classes. As shown in Supplementary Figure S2a, potency distributions in activity classes from medicinal chemistry are not uniform but skewed because most compounds are generally active in the low micromolar range. Therefore, we reasoned that the dominance of compounds with micromolar potency values might explain the strong performance of kNN and MR relative to SVM. Consequently, we generated activity class variants with balanced potency distributions (see Section 4), as shown in Supplementary Figure S2b. In the modified data sets, most potency sub-ranges were evenly populated (except sub-ranges containing limited numbers of most potent compounds). Thus, balancing eliminated the bias of potency value distributions towards the low micromolar range. We then repeated the predictions on the balanced activity class variants. Since balancing inevitably led to a reduction in data set size, we also generated equally sized data sets with original potency distribution as a control (50/50% training/test compound splits). Figure 3 reports the results for the predictions on balanced data sets that were similar to those of the original predictions. As a consequence of potency balancing, the median potency values of the training set increased, which also increased the MAE of MR in several cases. However, the performance of kNN/1-NN compared to SVR essentially remained constant.

### 2.4. Removal of Nearest Neighbors

**Figure 4:**
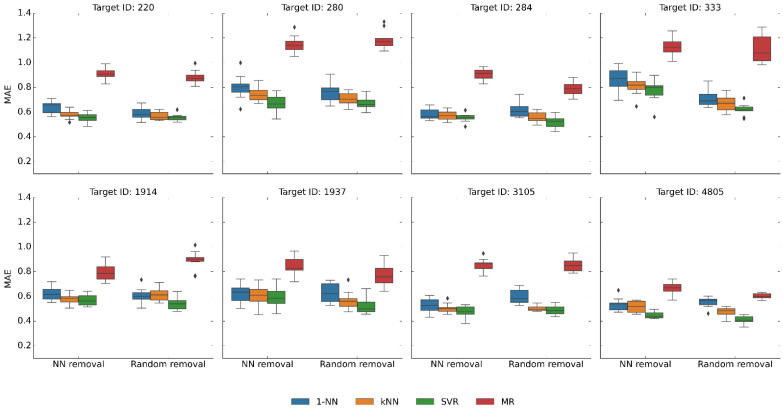
Prediction accuracy after removal of nearest neighbor relationships. Boxplots report the distribution of MAE values over 10 independent trials for the eight activity classes after removal of 50% of nearest neighbors and control data sets after random removal of 50% of the compounds. In boxplots, the upper and lower whiskers indicate maximum and minimum values, the boundaries of the box represent the upper and lower quartiles, values classified as statistical outliers are shown as diamonds, and the median value is indicated by a horizontal line.

In light of these findings, we systematically removed nearest neighbors from the original activity classes. Therefore, exhaustive pairwise compound similarity calculations were carried out for each class; compounds were ranked according to highest similarity to nearest neighbors, and the top 50% of compounds from the ranking were removed from the data sets. As a size control, data sets containing half of the original compounds were randomly selected. Figure 4 shows the results of predictions after nearest neighbor removal and equally sized control data sets (all 45 activity classes produced equivalent results). Nearest neighbor removal generally increased median MAE values for all methods by ~0.1–0.2 and slightly broadened value distributions (such that the predictions became again more similar to MR). However, even the removal of 50% of most similar compounds was insufficient to significantly reduce the performance of kNN/1-NN relative to SVR, an unexpected finding.

### 2.5. Analog Series-Based Data Partitioning

**Figure 5:**
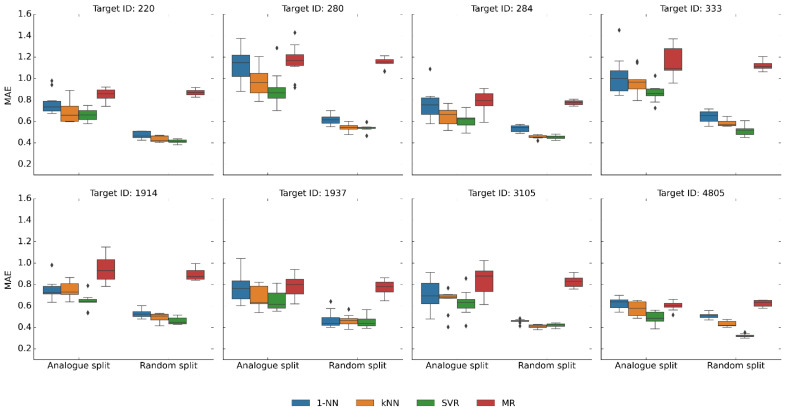
Prediction accuracy after analog series partitioning. Boxplots report the distribution of MAE values over 10 independent trials for the eight activity classes using training and test sets (~80:20% compound split) consisting of distinct analog series. As a control, results are reported for original training and test sets of exactly the same size. In boxplots, the upper and lower whiskers indicate maximum and minimum values, the boundaries of the box represent the upper and lower quartiles, values classified as statistical outliers are shown as diamonds, and the median value is indicated by a horizontal line.

Another structural data set modification was carried out by extracting all analog series from each activity class, then partitioning the complete series into training and test sets (to obtain ~80/20% compound splits). Accordingly, there was no analog overlap between the sets. Accordingly, training and test compounds had distinct core structures. Because most compounds from medicinal chemistry belong to analog series (resulting from chemical optimization efforts), analog series-based partitioning was generally applicable to activity classes. Figure 5 shows the results of predictions for these activity class variants and equally sized subsets of the original data sets used as a control (equivalent results were again obtained for all 45 activity classes). Under these conditions, median MAE values also increased by ~0.1–0.2 relative to the controls. The value distributions generally broadened (as one might expect for independent trials using training and test sets of unique analog series composition). Broader distributions are indicative of more variable (less stable) predictions, which complicates the comparison of different methods. However, despite analog series partitioning, the predictive performance of SVR and kNN/1-NN remained very similar.

## 3. Discussion

Our current study was designed in light of previous observations that simple 1-NN calculations often approached or met the accuracy of increasingly complex ML methods in compound potency predictions. To better understand these prediction characteristics and explore consequences for benchmark comparisons of different methods, we have carried out systematic potency value predictions on 376 activity classes with sufficient numbers of compounds using a preferred ML approach and simple controls, including kNN and MR calculations. Activity classes were curated to ensure that high-confidence activity measurements were available for all compounds, thus avoiding potential bias of predictions due to limited data quality. Our calculations most likely represent one of the largest (if not the largest) compound potency prediction campaigns reported to date. The results of the global predictions were surprisingly similar across a large number of activity classes from three points of view. First, there were only little activity class-specific differences in prediction patterns and accuracy; second, most predictions had limited error margins falling well within an order of magnitude; third, in accordance with our earlier observations, kNN calculations consistently rivaled SVR performance, and there was only a small error range separating prediction accuracy including MR, the most control. Thus, global potency predictions were surprisingly stable and accurate for methods of different complexity. These findings implied that calculations on activity classes from medicinal chemistry might generally produce predictions that are too similar for a realistic assessment and comparison of different potency prediction methods. Accordingly, the results also call the relevance of conventional benchmark settings into question. Benchmark calculations are essential for assessing basic method performance but must also reliably quantify relative differences in the accuracy of alternative approaches. Therefore, we then explored (i) possible reasons for the success of simple potency prediction approaches and (ii) ways in which activity classes and calculation conditions might be modified to increase the difficulty and sensitivity of benchmarking using model data sets. Specifically, we balanced potency distributions in activity classes, removed large numbers of nearest neighbors from them, and trained and tested models on structurally distinct compound sets obtained by analog series partitioning. Predictions on model data sets were again unexpectedly robust. Notably, while minor increases in prediction errors were observed for modifications rendering the predictions more challenging, none of these operations led to a significant difference in relative performance between SVR and kNN. The observed stability and robustness of the predictions on original and modified activity classes can be positively viewed because promising predictions are obviously possible with rather different approaches and using data set variants of varying composition. However, for conventional benchmarking, the implications are profound. Based on the findings reported herein, benchmark calculations on activity classes from medicinal chemistry, even if specifically modified to increase prediction challenges, do not enable sound comparisons of different methods because alternative predictions, including simple controls, are only differentiated by small error margins. A potential reason for this might include the prevalence of structurally related compounds with similar potency in activity classes (originating from chemical optimization efforts) or the under-representation of highly potent compounds in data sets (representing the most attractive prediction targets). As shown herein, however, predictions were resistant to substantial structural modifications of activity classes. Thus, from this point of view, our study should raise awareness of these issues and trigger attempts to develop fundamentally different concepts for evaluating and comparing potency prediction methods, providing opportunities for future investigations.

## 4. Materials and Methods

### 4.1. Compound Activity Data

From ChEMBL release 30 [13], bioactive compounds of less than 1000 Da with standard potency measurements (IC_50_) and a numerical specified potency value (standard relation ‘=’) were retrieved. Potency values were recorded as the negative decadic logarithm. Only compounds with direct interactions (target relationship type: “D”) against human proteins at the highest confidence level (target confidence score: 9) and pIC_50_ values ranging from 5 to 11 were considered. Additionally, measurements labeled “potential transcription error” and “potential author error’’ were removed. In addition, potential assay interference compounds were removed using public filters and tools [14,15,16].

Based on these selection criteria, 91,733 compounds belonging to 376 activity classes containing at least 50 compounds were obtained for the analysis. The largest 45 activity classes consisted of at least 500 compounds each (yielding 40,440).

### 4.2. Compound Sets with Balanced Potency Distribution

The 45 largest activity classes were balanced to obtain an even potency value distribution across the entire potency range, yielding reduced data sets of 50% of the original size. These balanced data sets were generated by dividing the potency range of each class into a maximum of six equally sized bins (for logarithmic potency values of 5–6, 6–7, 7–8, 8–9, 9–10, and 10–11). The average number of compounds per bin was calculated by dividing the number of available compounds by the number of bins. The bins were subsequently populated with compounds until the number was equal to the calculated average. For bins representing highest potency values, the number of available compounds was often insufficient to satisfy this criterion. In this case, other potency bins for which compounds were still available were uniformly populated until the final size of the balanced set was equal to 50% of the original compound set.

### 4.3. Model Building and Implementation

For model building and evaluation, training and test sets were generated using random and analog series-based compound partitioning. For each activity class, compounds were randomly partitioned to obtain 80/20% and 50/50% training/test compound splits. In addition, analog series comprising at least two compounds were systematically extracted from activity classes using the compound–core relationship algorithm [17]. Remaining singletons were discarded. The analog series were then partitioned into training and test sets corresponding to ~80/20% training/test compound splits such that both sets consisted of unique analog series with no analog overlap between sets.

#### 4.3.1. Support Vector Regression

SVR is an extension of the support vector machine algorithm for supervised learning that derives a regression function through the generation of an ε-insensitive tube using training data. If a linear data separation is not feasible in the original feature space, a kernel function is employed to project the data to a high-dimensional space where linear separation might become possible [7,8]. For SVR, the regularization hyper-parameter C was determined by testing (0.001, 0.005, 0.01, 0.05, 0.1, 0.5, 1, 10, 100, and 10,000) values. SVR models were derived using the Tanimoto kernel [18].

#### 4.3.2. k-Nearest Neighbor Regression

kNN is a non-parametric supervised learning method that ranks training compounds based on increasing molecular similarity (decreasing distance). For a test compound, the potency is then determined based on the potency values of the k top-ranked compounds from the training set [19]. For kNN, the best-performing k values were determined for one, three, and five top-ranked compounds by averaging potency values for three and five compounds. In addition to applying optimized kNN values, 1-NN predictions were consistently reported for all activity classes. For compound comparison, Tanimoto similarity was calculated using the folded 2048-bit version of the extended connectivity fingerprint with bond diameter 4 (ECFP4) [20] generated with RDKit [21].

#### 4.3.3. Median Regression

The MR control calculation uniformly assigns the median potency value of the training set to each test set compound. This approach was employed as a control calculation.

#### 4.3.4. Hyperparameter Optimization

For parameter optimization, kNN and SVR were submitted to a grid search with 5-fold internal cross-validation implemented using scikit-learn [22].

### 4.4. Molecular Representation

For modeling, compounds were represented using the folded 2048-bit version of ECFP4 generated using RDKit.

### 4.5. Performance Metric

To evaluate model performance, the mean absolute error (MAE) was calculated by comparing predicted and experimental test compound potency values. The calculations were carried out using scikit-learn. MAE is defined as

(1) $$MAE\left(y,\hat{y}\right)=\frac{1}{n}\overset{n}{\underset{i=1}{\sum }}\left|y_{i}-{\hat{y}}_{i}\right|$$

where *n* is the number of compounds, and *y* and *ŷ* are the experimental and predicted potency values, respectively.

Increasing MAE values indicate decreasing prediction accuracy and vice versa.

### 4.6. Statistical Significance Testing

Statistical significance evaluation of differences between MAE value distributions was carried out using the Wilcoxon test [23]. The alpha value with Bonferroni correction (*n* = 10) was set to 0.005 and compared to the respective *p*-value (*p* < 0.005).

## 5. Conclusions

In this work, we have systematically investigated compound potency predictions on nearly 400 different activity classes using ML and simple control models. In accord with earlier observations, methods of different complexity produced overall similar prediction accuracy differentiated by only small error margins, as demonstrated now on a very large scale. Moreover, relative method performance remained stable despite specific potency range and structural data set modifications designed to increase the difficulty of the calculations. Taken together, our findings clearly indicate that conventional benchmark calculations are not a realistic indicator of differences in the predictive performance of alternative computational methods. Therefore, future research in this area should focus on exploring and devising new concepts for benchmarking potency prediction methods.

## Data Availability

Data is contained within the article and supplementary material.

## Supplementary Materials

The following supporting information can be downloaded at: https://www.mdpi.com/article/10.3390/ph16040530/s1, Figure S1: Prediction accuracy, Figure S2: Potency value distributions.
