# Supplementary material for: Large-Scale Predictions of Compound Potency with Original and Modified Activity Classes Reveal General Prediction Characteristics and Intrinsic Limitations of Conventional Benchmarking Calculations
Source: Pharmaceuticals (Basel). 2023 Apr 2;16(4):530. doi: 10.3390/ph16040530 (PMC10143224; doi:10.3390/ph16040530)
Supplement: Supplementary file 1 [file pharmaceuticals-16-00530-s001.zip › pharmaceuticals-2320370-supplementary.pdf]

Supplementary Materials

a

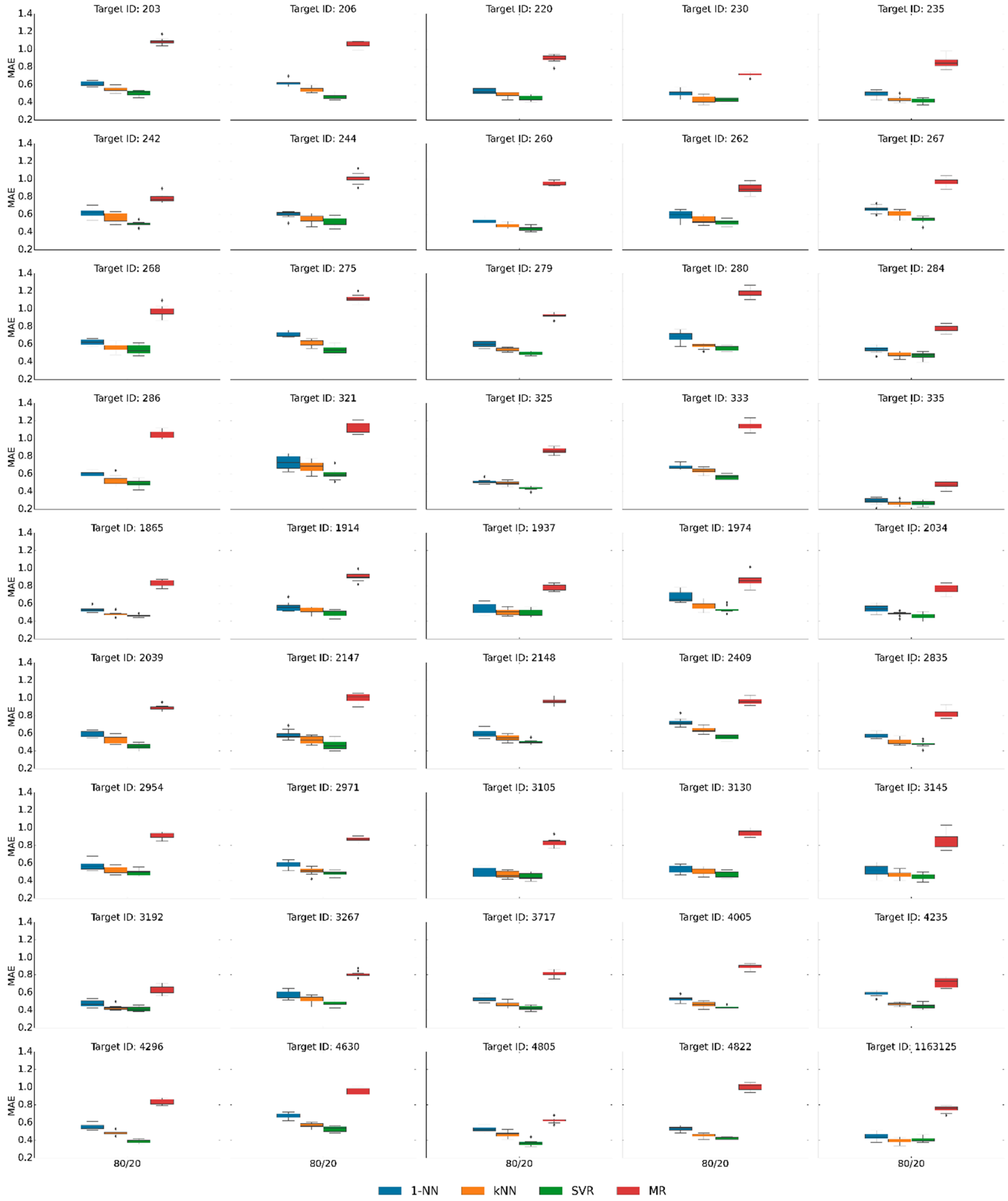

b

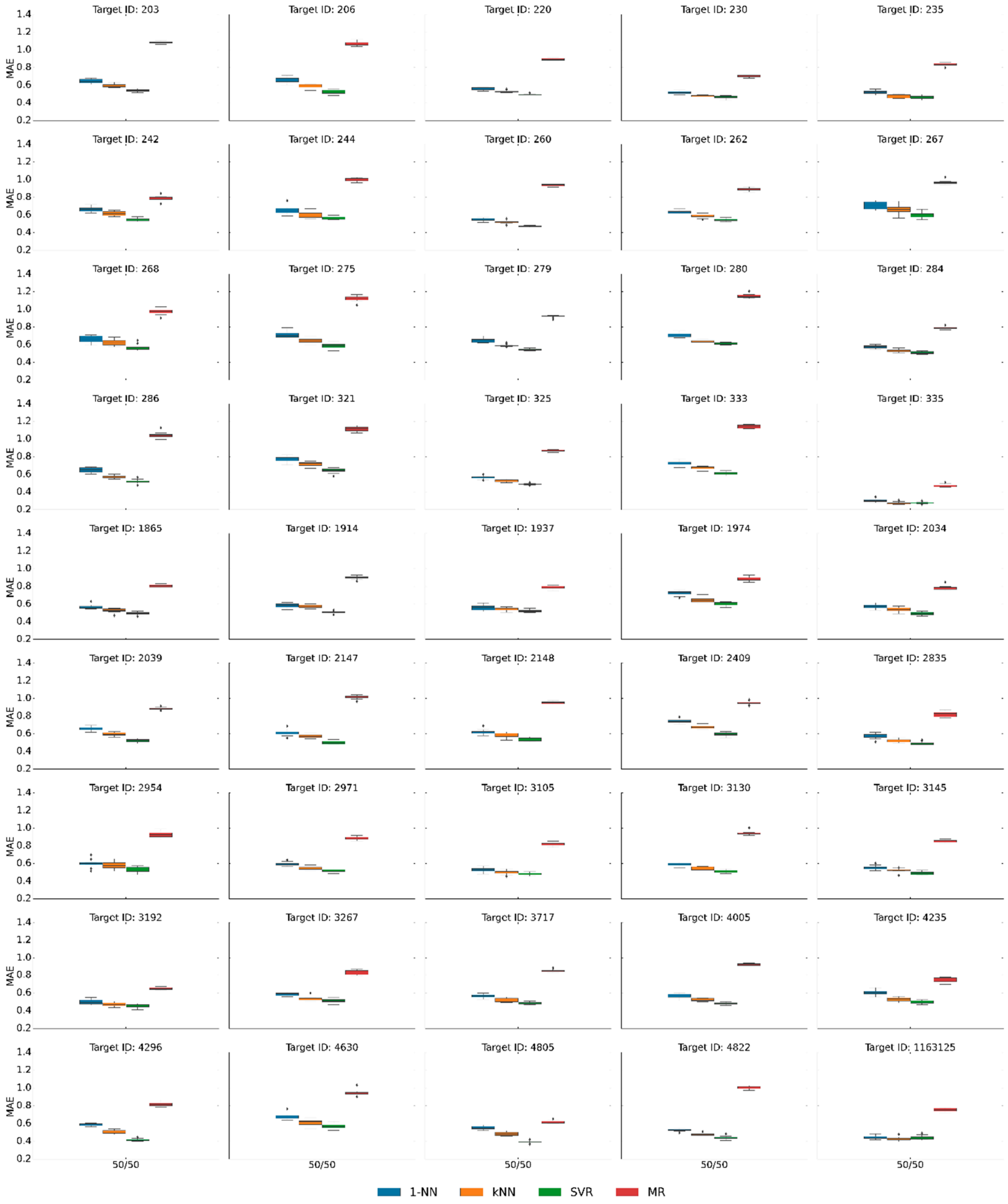

Figure S1. Prediction accuracy. Boxplots report the distribution of MAE values for potency predictions over 10 independent trials on 45 activity classes using 1NN, kNN, SVR, and MR models for training/test set compound splits of (**a**) 80:20% and (**b**) 50:50%.

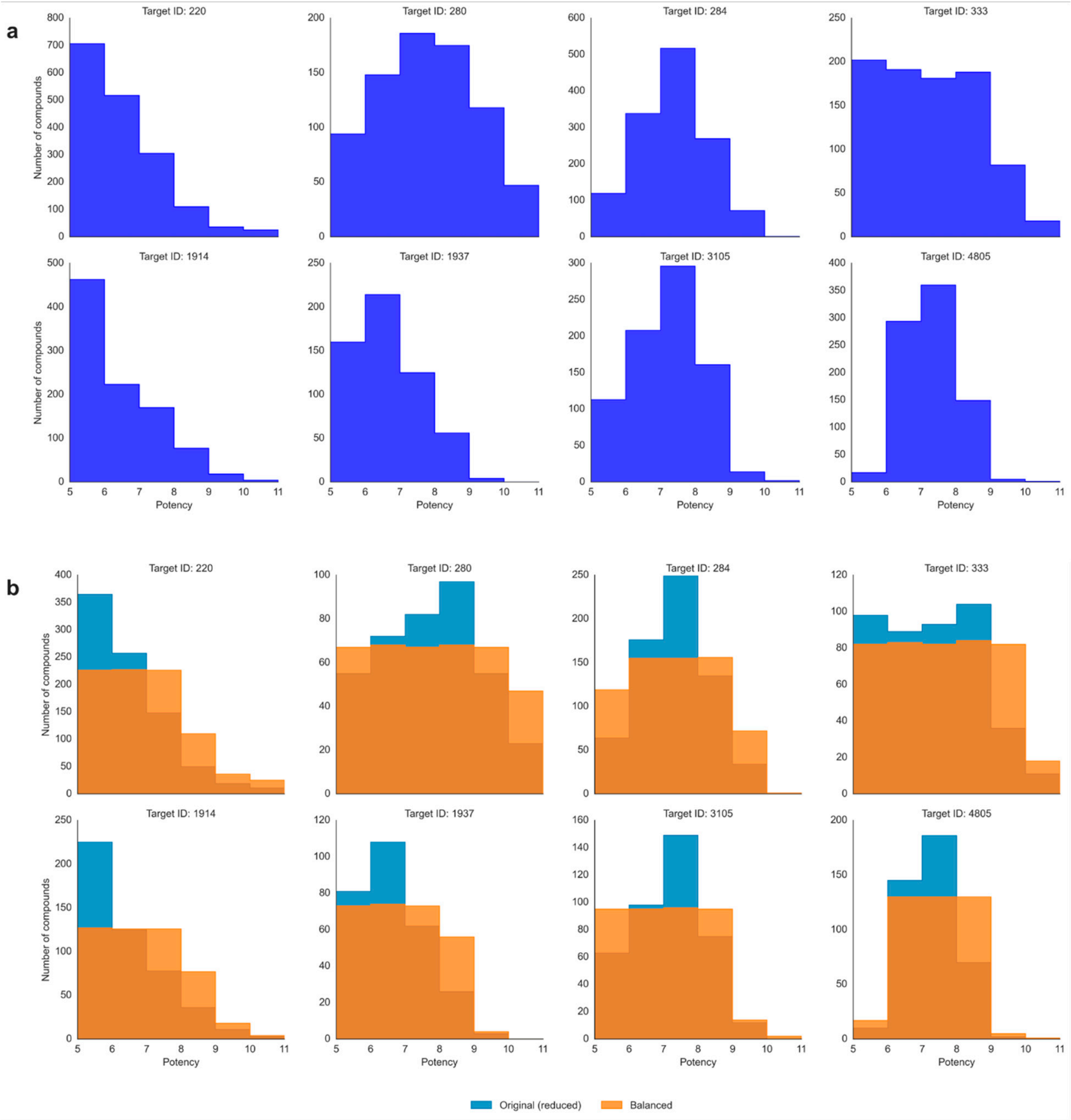

Figure S2. Potency value distributions. For the eight activity classes in Figure 1, binned potency value distributions are shown (**a**) before and (**b**) after balancing.
